# Small Airways Dysfunction and Remission in Adults With Asthma: A Longitudinal Exploratory Analysis of the AssessmenT of smalL Airways involvemeNT In aSthma (ATLANTIS) Study

**DOI:** 10.1111/all.70264

**Published:** 2026-02-18

**Authors:** Akshi Kumar, Rory Chan, Nazanin Zounemat‐Kermani, Eleanor Quek, Ian M. Adcock, Bianca Beghe, Christopher Brightling, Dave Singh, Janwillem Kocks, Alberto Papi, Klaus F. Rabe, Ulrica Scaffidi‐Argentina, Maarten van den Berge, Monica Kraft, Salman Siddiqui

**Affiliations:** ^1^ National Heart and Lung Institute, NIHR Imperial Biomedical Research Centre, Imperial College London London UK; ^2^ School of Medicine University of Dundee Dundee UK; ^3^ Data Science Institute, Imperial College London London UK; ^4^ Respiratory Unit, Hospital Policlinico Modena, Department of Surgical and Medical Sciences University of Modena and Reggio Emilia Modena Italy; ^5^ Institute for Lung Health, National Institute for Health Research Biomedical Research Centre University of Leicester Leicester UK; ^6^ Centre for Respiratory Medicine and Allergy University of Manchester, Manchester University NHS Foundation Trust Manchester UK; ^7^ General Practitioners Research Institute Groningen the Netherlands; ^8^ Department of Translational Medicine University of Ferrara Ferrara Italy; ^9^ LungenClinic Grosshansdorf Grosshansdorf Germany; ^10^ Airway Research Center North, German Center for Lung Research Grosshansdorf Germany; ^11^ Christian‐Albrechts University of Kiel Kiel Germany; ^12^ Global Medical Affairs Chiesi Farmaceutici S.p.A. Parma Italy; ^13^ Department of Pulmonary Diseases University of Groningen, University Medical Center Groningen Groningen the Netherlands; ^14^ Department of Medicine, Icahn School of Medicine at Mount Sinai Mount Sinai Health System New York New York USA

**Keywords:** asthma, disease exacerbation, factor analysis, gene expression analysis, remission induction, spontaneous remission

## Abstract

**Background:**

Asthma remission is a feasible treatment goal. However, remission definitions vary, and predictive biomarkers remain underexplored.

**Methods:**

We conducted a post hoc analysis of ATLANTIS (NCT02123667), a multinational prospective study including 684 adult asthmatics. Remission was defined by 3‐component (3C) and 4‐component (4C) criteria. 3C remission included: (1) ACQ‐6 < 1.5, (2) no maintenance oral corticosteroids, (3) no exacerbations. An absolute decline < 10% in pre‐bronchodilator FEV_1_% predicted, was added for the 4C definition. Multivariate logistic regression identified remission predictors. A novel Low Disease Activity (LDA) score was developed using factor analysis of five clinical variables (ACQ‐6, FeNO, BEC, and FEV_1_) including an innovative small airways dysfunction questionnaire tool (SADT). Nasal transcriptomics were analysed for differential gene expression and pathway enrichment and were replicated in U‐BIOPRED (NCT01976767) using sputum transcriptomics. U‐BIOPRED was included only to study omics replication of remission pathways identified in ATLANTIS.

**Findings:**

Remission occurred in 48% (3C) and 45% (4C) of patients. Predictors included male sex, better lung function, fewer previous exacerbations, and higher SADT (fewer small airways symptoms). LDA identified milder disease and was associated with remission [OR 3C 4.43 (2.80, 7.10) and 4C 3.46 (2.23, 5.43)], improved QoL [OR 2.07 (1.65, 2.60)], and fewer future exacerbations [OR 0.43 (0.22, 0.85)]. Transcriptomic analyses revealed remission‐associated upregulation of interleukin 4/13 signalling and downregulation of coagulation pathways, in both ATLANTIS and U‐BIOPRED.

**Interpretation:**

SAD was associated with reduced asthma remission. A novel LDA tool demonstrated clinical utility in stratifying prospective asthma risk. Key immunologic and haemostatic pathways may underpin remission, offering potential targets for future intervention.

## Graphical abstract

We conducted a post hoc analysis of ATLANTIS, a multinational prospective study including 684 adults with asthma. Fewer small airway symptoms and lower disease activity are independently associated with asthma remission. Replicated airway transcriptomic signatures show increased IL‐4/13 signalling and reduced coagulation pathways across the ATLANTIS and U‐BIOPRED cohorts. ACQ‐6, asthma control questionnaire‐6; ATLANTIS, assessment of small airways involvement in asthma; BEC, blood eosinophil count; BMI, body mass index; FeNO, fractional exhaled nitric oxide; FEV_1_ , forced expiratory volume in 1 s; LDA, low disease activity; mOCS, maintenance oral corticosteroids; Pre‐BD FEV_1_ , pre‐bronchodilator forced expiratory volume in 1 s % predicted; SADT, small airways dysfunction tool; U‐BIOPRED, unbiased biomarkers for the prediction of respiratory disease outcomes.

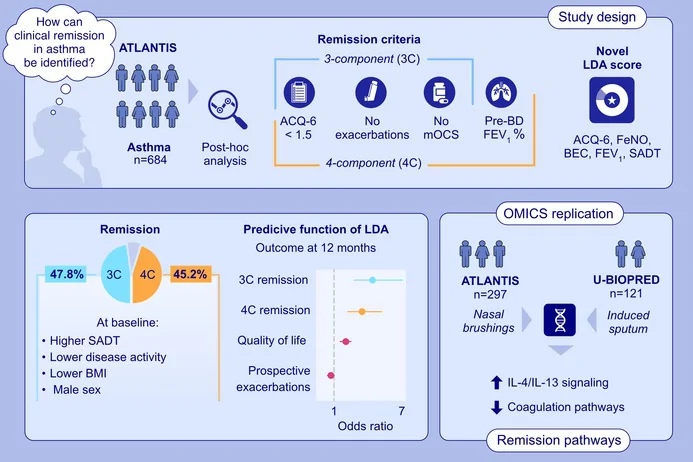

## 1 Introduction

Asthma is a chronic inflammatory airways condition, marked by recurring inflammation, increased mucus production, and heightened airway sensitivity, affecting up to 29% of the population [1]. Around 5%–10% of the asthma population suffer from severe, treatment‐resistant asthma and may experience persistent, uncontrolled inflammatory episodes [1]. These changes can progress to pathological structural alterations [2] such as collagen deposition, goblet cell hypertrophy, subepithelial fibrosis, and smooth muscle hypertrophy and hyperplasia, ultimately leading to largely irreversible airway remodelling and lung function decline. Small airways dysfunction (SAD) is present in all stages of asthma and associated with asthma exacerbations [3], exercise limitation, airway hyper responsiveness [4] and more severe asthma [5].

Growing interest in remission as a clinically relevant outcome has emerged alongside advances in asthma therapeutics. In recent years, a new class of treatments known as biologics has demonstrated remarkable effectiveness in selected patients with previously refractory asthma, considerably reducing exacerbation frequency, symptoms, and improving quality of life and lung function [6]. This has prompted a shift in focus toward remission as a more ambitious and attainable treatment goal. While the concept of remission is not entirely new, it is well established in fields such as rheumatology [7], oncology [8], and other disciplines [9].

Recognising remission as an achievable goal could prompt a shift in clinical guidelines from the traditional ‘treat‐to‐fail’ model to a more proactive ‘treat‐to‐target’ strategy [10]. This approach, already established in the management of rheumatoid arthritis [11], and applied in conditions like diabetes and hypertension [12], focuses on setting a clear, long‐term objective such as remission and actively guiding patients toward it. It also simplifies treatment by aligning management around a singular goal and addressing individual treatable traits, thereby enhancing patient engagement. While achieving full remission may not be feasible for all patients, an alternative target such as ‘low disease activity’ (LDA) can be set. In this regard, disease activity can be defined as a reversible component of disease, linked to the pathophysiology of the disease [13]. Although less clearly defined, LDA emphasises improved quality of life and a reduced disease burden, offering a more realistic and incremental goal as previously defined for rheumatoid arthritis [14]. Patients can then aim to progress toward full remission over time. Overall, this strategy embraces asthma's heterogeneity, supports patient‐centred care, and promotes proactive, tailored disease management [10].

Proposed predictors of remission include male sex [15], milder disease [16], shorter disease duration [15], younger age [17], lower airway hyperresponsiveness (AHR) [17], non‐smoking status [15], and lower BMI [15]. Inflammatory markers such as elevated fractional exhaled nitric oxide (FeNO) [18], increased sputum eosinophils, and reduced neutrophils [19] have also been associated with remission. Recent evidence also suggests that various extrapulmonary risk factors contribute to asthma non‐remission, indicating a potential need for earlier intervention in the disease course [20].

Based on this, we hypothesised that SAD, clinical characteristics, and inflammatory biomarkers could significantly predict asthma remission. Additionally, we aimed to examine the relationship between LDA, including SAD, with both baseline and longitudinal clinical outcomes in an exploratory analysis. Lastly, we investigated the association between dysregulated genes and molecular pathways with remission status. Our goals included determining how clinical remission in asthma can be identified, characterised, and mechanistically understood.

## 2 Materials and Methods

The ATLANTIS (AssessmenT of smalL Airways involvemeNT In aSthma) study (NCT02123667) study, a multinational, prospective, observational study was conducted between 2014 and 2017 across 29 academic medical centres [3]. All participants were over 18 years old and had a clinical diagnosis of asthma for at least 6 months before the baseline visit, in accordance with the GINA 2012 staging criteria. Subjects attended three clinical visits: at baseline, 6 months, and 12 months. Our replication cohort for omics consisted of adults from the Unbiased Biomarkers for the Prediction of Respiratory Disease Outcomes (U‐BIOPRED) study (NCT01976767), a multinational, prospective, observational study conducted between 2010 and 2015 across 16 clinical centers [21]. All participants underwent a baseline visit, and individuals with severe asthma were followed up at 12 months. For inclusion in this study, patients were required to attend both visits. Detailed methodological information can be found in the supplementary appendix of a previous ATLANTIS publication [3].

Our chosen definitions of remission were: (1) 3C Remission: ACQ‐6 < 1.5, no maintenance oral corticosteroids (mOCS) for disease control and no exacerbations, and (2) 4C Remission: ACQ‐6 < 1.5, no mOCS for disease control, no exacerbations, and an absolute decline in pre‐bronchodilator FEV_1_% predicted < 10%. Criteria must be met at all timepoints throughout the 52 weeks.

Patients were included in this post hoc analysis if they met the screening criteria [3], completed all visits, and provided full follow‐up data for all remission criteria. For the LDA analysis, only patients with complete follow‐up data for all assessed variables were included. A complete dataset was provided in spreadsheet format prior to analysis. Data including questionnaires, clinical records, and imaging, were analysed for our remission and LDA assessments [3]. For genetic analysis, RNA sequencing of nasal brushings from the ATLANTIS study and proteomic profiling of induced sputum samples from U‐BIOPRED were evaluated [3, 21].

Each analysis focused on specific subsets determined by the presence of missing data. Baseline characteristics were evaluated using the Mann–Whitney *U* test, Chi‐squared test, or Fisher's exact test, depending on the distribution and type of variable. Significant variables were then included in multivariate logistic regression models. Model performance was assessed using Receiver Operating Characteristic (ROC) curves as area under the curve (AUC), and the Akaike Information Criterion (AIC).

To investigate LDA, an exploratory standardised factor analysis was performed using five variables (ACQ‐6, FeNO, SADT, BEC, and pre‐bronchodilator FEV_1_) to derive composite disease activity scores. A higher SADT score indicates less SAD whereas a higher small airway physiology score relates to more SAD [22].

Lower scores reflected lower disease activity, with the lowest quartile classified as LDA. Baseline LDA status was then compared against both baseline and longitudinal clinical outcomes. Statistical significance for remission and LDA analyses was defined as *p* < 0.05. Definitions for LDA, SAD, SADT, and SAD physiological score can be found in the Supporting Information (supplementary methods section). Additional details on the factor analysis are provided in Table S1.

Principal Component Analysis (PCA) and Differential Gene Expression (DGE) were carried out on 3‐component (3C) and 4‐component (4C) remission. The significant genes identified through DGE are presented in Tables S2–S4. Dysregulated genes entered Pathway Enrichment Analysis (PEA). Dysregulated genes had a log (Fold Change) > 0.5 or < −0.5, whilst two significance levels were set: *p* < 0.05 and *p* < 0.01 (Tables S5–S7). Unadjusted *p* values are used, as correction for False Discovery Rate with Benjamin‐Hochberg rendered no results.

All statistical analyses were conducted through R Statistical Software, Version 2023 12.0+369 (Posit Software, PBC, Boston, MA.). Differential Gene Expression was conducted with DESeq2 (1.42.1) with a negative binomial generalised linear model. Differential protein analysis was conducted using the limma package (v3), applying linear models with empirical Bayes moderation. Factor analysis was conducted with Psych (2.4.3) with Ordinary Least Squares method to generate minimum residual solution. A full list of R packages can be accessed in Supporting Information. Pathway enrichment analysis was conducted through Reactome databases, 2022 by Posit Software, PBC. Statistical software references are provided in the Supporting Information (supplementary methods section). The Medical Ethics Committee of each centre approved the protocol, and all patients gave their written informed consent.

## 3 Results

**FIGURE 1:**
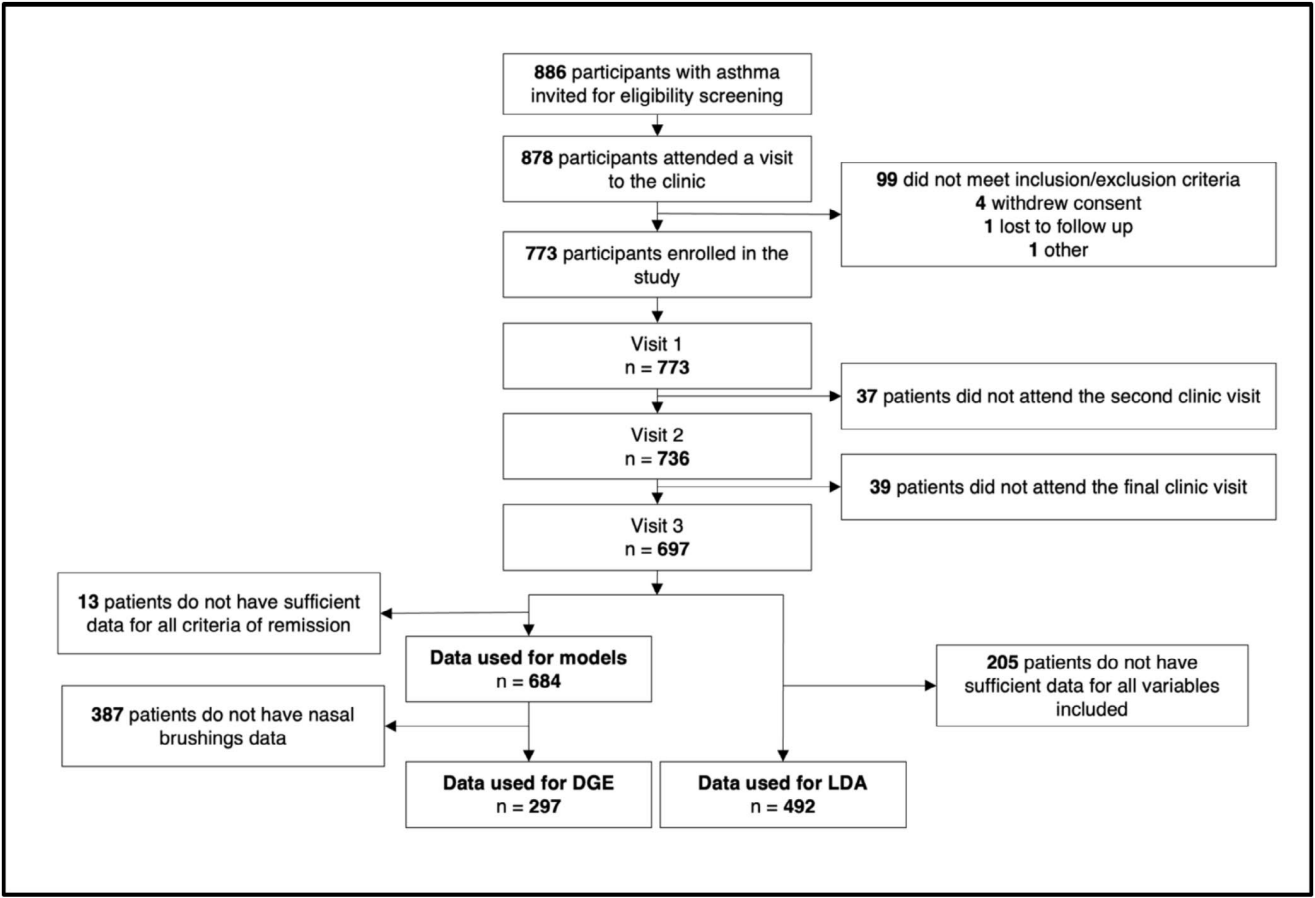
CONSORT diagram of the ATLANTIS discovery cohort. Participants were included only if they had complete follow‐up and full data for all four components of 4‐component remission (Asthma Control Questionnaire‐6 < 1.5, no maintenance oral corticosteroids for disease control, no exacerbations and a pre‐bronchodilator forced expiratory volume in 1 s (FEV_1_) % predicted absolute decline < 10%), at all timepoints throughout the study. Missing data were excluded on a variable‐by‐variable basis. DGE, differential gene expression; LDA, low disease activity.

The participant CONSORT diagram is depicted in Figure 1. Among 684 participants, 327 (47.8%) satisfied the criteria for 3C remission, while 309 (45.2%) satisfied the criteria for 4C remission. At baseline, patients in remission were typically younger, had a shorter disease duration, experienced fewer prior exacerbations, had a lower BMI, and were predominantly male. They also showed a higher prevalence of mild‐to‐moderate asthma, required lower doses of mOCS, and reported better scores on the ACQ‐6, ACT, and Euro‐QoL questionnaires. Additionally, patients in remission had lower physiological scores for SAD, higher SADT scores, and lower levels of BEC and BNC, and sputum neutrophil percentage (Neu%) (Table 1). In the continuous analyses, log‐transformed FeNO (*p* = 0.130) and log‐transformed BEC (*p* = 0.383) were not significantly associated with the outcome, and the interaction between logFeNO and logBEC was also not statistically significant (*p* = 0.104). Of the 223 participants in our U‐BIOPRED severe asthma subset, 7 (3.1%) met the criteria for 3C remission, and 3 (1.3%) met the criteria for 4C remission. The CONSORT diagram for U‐BIOPRED is presented in Figure S1.

**TABLE 1:** Baseline demographic, clinical, and inflammatory characteristics of remission and non‐remission participants.

Variable	Categories	Non‐remission	*n*	Remission			*n*	*p*	
				3cR (*n* = 327)	*n*	4cR (*n* = 309)		3cR	4cR
Baseline age		48 (35–56)	357	45 (33.5–53)	327	45 (34–53)	309	**0.034**	**0.027**
Age at diagnosis		23.41 (8.00–41.8)	357	24.9 (10.0–40.5)	327	24.7 (10.0–40.2)	309	0.564	0.732
Duration of disease		19.0 (6.22–29.6)	356	15.0 (5.40–28.2)	327	14.7 (5.34–28.7)	309	**0.025**	**0.029**
Prior exacerbations (last 12 months)		0.317 (±0.795)	357	0.070 (±0.320)	327	0.068 (±0.320)	309	**< 0.001**	**< 0.001**
Exacerbations during the study		1.26 (±2.05)	357	0.00 (±0.00)	327	0.00 (±0.00)	327	**< 0.001**	**< 0.001**
Body‐mass index (kg/m^2^)		26.7 (23.6–30.5)	357	25.4 (22.8–29.0)	327	25.4 (22.8–28.9)	309	**0.001**	**< 0.001**
Sex			357		327		309	**< 0.001**	**< 0.001**
	Female	239 (67.0%)		156 (47.7%)		147 (47.6%)			
	Male	118 (33.0%)		171 (52.3%)		162 (52.3%)			
GINA categories			357		327		309	**< 0.001**	**< 0.001**
	GINA 1	46 (12.8%)		74 (22.6%)		70 (22.7%)			
	GINA 2–3	101 (28.3%)		150 (45.9%)		141 (45.6%)			
	GINA 4–5	210 (58.8%)		103 (31.5%)		98 (31.7%)			
	GINA 5 + mOCS	16		0		0		**< 0.001**	**< 0.001**
Median mOCS dose (equivalent to Prednisone)		5 (5–15)	357	0 (0–0)	327	0 (0–0)	309	**< 0.001**	**< 0.001**
Smoking status			357		327		309	0.970	0.980
	Current Smoker	13 (4.5%)		13 (4.5%)		12 (3.9%)			
	Ex‐smoker	71 (24.5%)		66 (22.7%)		61 (19.7%)			
	No smoking history	273 (94.5%)		248 (90.9%)		236 (76.3%)			
Symptom scores			357		327		309		
	ACQ‐6	1.33 (0.67–2.16)		0.33 (0.00–0.67)		0.33 (0.00–0.67)		**< 0.001**	**< 0.001**
	ACT	19 (16–22)		23 (21–25)		23 (21–25)		**< 0.001**	**< 0.001**
	Euro‐QOL	5.27 (4.40–6.00)		6.33 (5.80–6.73)		6.20 (5.53–6.60)		**< 0.001**	**< 0.001**
Pre‐bronchodilator FEV_1_% pred.		78.7 (65.3–90.2)	357	85.7 (74.5–95.4)	327	85.6 (74.3–94.8)	309	**< 0.001**	**< 0.001**
Post‐bronchodilator FEV_1_% pred.		87.3 (75.1–98.2)	357	94.3 (84.3–102)	327	94.5 (85.1–102)	309	**< 0.001**	**< 0.001**
FEV_1_/FVC		0.691 (0.610–0.760)	357	0.690 (0.631–0.763)	327	0.694 (0.640–0.764)	309	0.210	0.190
SADT questionnaire score		4.90 (± 1.41)		5.11 (± 1.34)		5.11 (± 1.34)		**0.007**	**0.008**
SAD Physiological Score		−0.02 (−0.26–0.37)	357	−0.08 (−0.30–0.09)	327	−0.08 (−0.30–0.08)	309	**< 0.001**	**< 0.001**
Blood neutrophil count (×10^9^ L^−1^)		3.79 (3.05–4.80)	354	3.53 (2.82–4.47)	325	3.52 (2.82–4.42)	308	**0.011**	**0.007**
Sputum neutrophils %		56.2 (35.5–74.8)	109	44.45 (25.03–62.23)	104	44.2 (24.2–61.38)	94	**0.015**	**0.013**
Sputum eosinophils %		0.60 (0.00–3.30)	109	0.30 (0.10–1.90)	104	0.30 (0.10–2.1)	94	0.386	0.337
Blood eosinophil count (×10^9^ L^−1^)		0.26 (0.14–0.40)	354	0.22 (0.13–0.34)	325	0.21 (0.13–0.34)	308	**0.039**	**0.031**
Blood eosinophil count (×10^9^ L^−1^)			354		325		308	0.097	0.072
	< 0.15	103 (29.1%)		111 (34.1%)		107 (34.7%)			
	0.15–0.30	103 (29.1%)		104 (32.0%)		98 (31.8%)			
	> 0.30	148 (41.8%)		110 (33.8%)		103 (33.4%)			
FeNO (ppb)		23 (15–37)	284	26 (17–38)	276	26 (17–39)	261	0.067	0.107
FeNO (ppb)			284		276		261	0.132	0.114
	< 25	164 (57.7%)		140 (50.7%)		132 (50.6%)			
	25–50	74 (26.1%)		93 (33.7%)		89 (34.1%)			
	> 50	46 (16.2%)		43 (15.6%)		40 (15.3%)			

*Note:* Characteristics of participants stratified by remission status over 52 weeks. Data are presented as mean ± standard deviation, median (interquartile range), or *n* (%), as appropriate. Normality was assessed using the Kruskal–Wallis test. Categorical variables were analysed using Chi‐squared or Fisher's exact test. Continuous variables were compared using Mann–Whitney *U* test or Student's *t*‐test, depending on data distribution. Bold values denote statistical significance with a two tailed *p* < 0.05.

Abbreviations: 3cR, 3 component remission; 4cR, 4 component remission; ACQ, asthma control questionnaire; BMI, body mass index; FeNO, fractional exhaled nitric oxide; GINA, global initiative for asthma; ppb, parts per billion; SAD, small airways dysfunction; SADT, small airways dysfunction tool.

**FIGURE 2:**
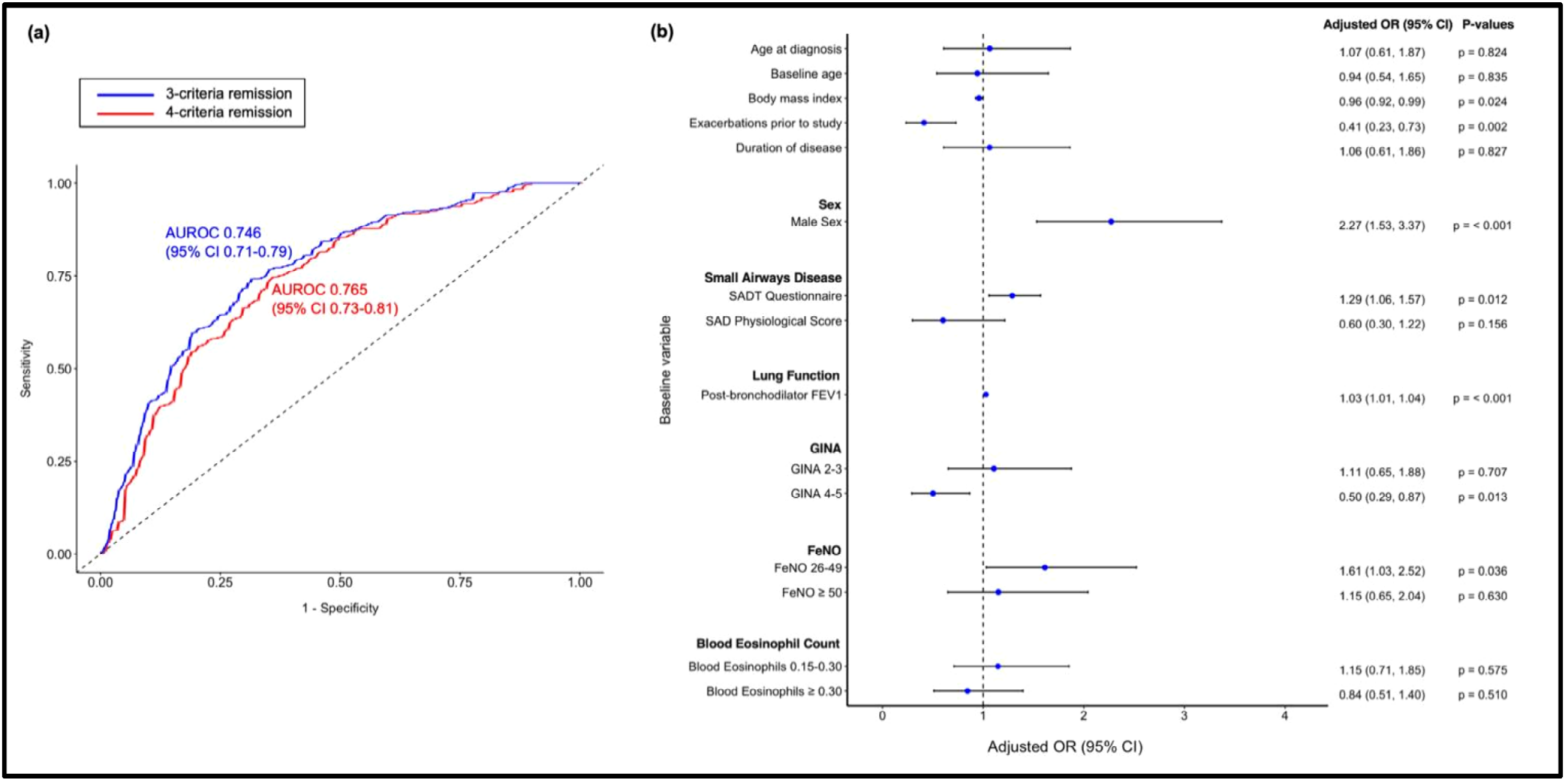
(a) Receiver Operating Characteristic (ROC) curves comparing the predictive performance of the ATLANTIS models for 3‐component and 4‐component clinical remission. Area under the ROC curve (AUROC) values with 95% confidence intervals are presented for both models. (b) Forest plot illustrating the results of a multivariable logistic regression model assessing baseline predictors of 3‐component remission in the ATLANTIS cohort. Variables were selected based on baseline characteristics and T2 biomarker profiles. The Small Airways Dysfunction Tool (SADT) is a questionnaire that reports lower levels of small airways dysfunction as higher questionnaire scores. In contrast, the Small Airways Dysfunction (SAD) Physiological Score is an objective marker of small airways dysfunction using physiological tests, which reports lower levels of small airways disease as lower scores. Odds ratios (OR) are displayed with 95% confidence intervals (95% CI). BMI, body mass index; FeNO, fractional exhaled nitric oxide; GINA, global initiative for asthma; Post‐BD FEF_25–75_% predicted, post‐bronchodilator forced expiratory flow at 25%–75% of full vital capacity; Post‐BD FEV_1_, post‐bronchodilator forced expiratory volume in 1 s % predicted; ppb, parts per billion; SAD Physiological Score, small airways dysfunction physiological score; SADT, small airways dysfunction tool.

Two multivariate logistic regression models were developed to identify key predictors of remission based on both sets of remission criteria. ROC curve analysis confirmed the models' ability to distinguish between outcomes, yielding AUC values of 0.75 (0.71, 0.79) for 3C remission and 0.77 (0.73, 0.81) for 4C remission (Figure 2a). Seven significant predictors of remission were identified (Figure 2b). Male sex emerged as the strongest predictor for achieving both 3C [OR 2.27 (1.53, 3.37)] and 4C remission. Higher SADT scores indicative of less small airways dysfunction related symptoms [OR 1.29 (1.06, 1.57)], moderate (25–50 ppb) FeNO levels [OR 1.61 (1.03, 2.52)], and better baseline lung function [OR 1.03 (1.01, 1.04)] were associated with increased chances of remission. Conversely, a history of frequent exacerbations [OR 0.41 (0.23, 0.73)], more severe asthma [OR 0.50 (0.29, 0.87)], and elevated BMI [OR 0.96 (0.92, 0.99)] were linked to a reduced likelihood of remission.

We assessed multicollinearity using the generalised variance inflation factor (GVIF) (Table S8). The analysis showed that most covariates, including BMI, sex, exacerbation history, symptom scores, lung function, inflammatory categories, and GINA group, had low GVIF values, indicating minimal collinearity and independent contributions to the model. In contrast, age, age at diagnosis, and disease duration exhibited high GVIF values, reflecting substantial overlap between these variables. This is expected given their inherent mathematical and clinical relationship (current age = age at diagnosis + disease duration).

**FIGURE 3:**
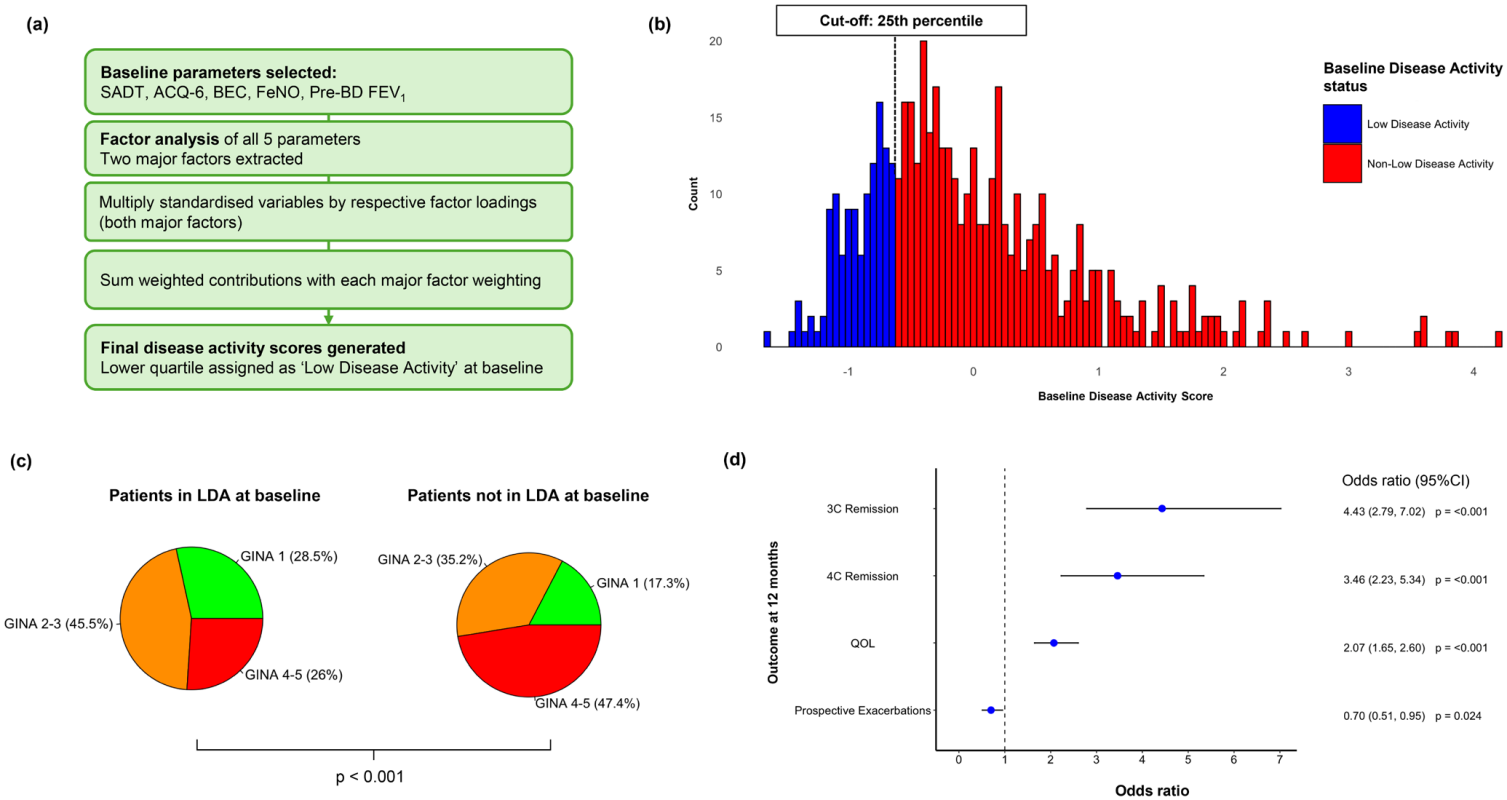
(a) Flow diagram demonstrating how the Low Disease Activity (LDA) scores were calculated through a factor analysis function. (b) Histogram of all LDA scores at baseline, showing distribution and the selected 25th percentile as the cut‐off, with lower scores being denoted as being in LDA at baseline. (c) Pie charts showing distribution of GINA classes of severity among two groups, those in LDA at baseline, and those not in LDA at baseline. (d) Forest plot reporting the predictive function of LDA at baseline on 4 distinct outcomes at 12 years—3 component remission; 4 component remission; Quality of Life (QOL) as measured on the Asthma Quality of Life Questionnaire (AQLQ); prospective exacerbations.

**FIGURE 4:**
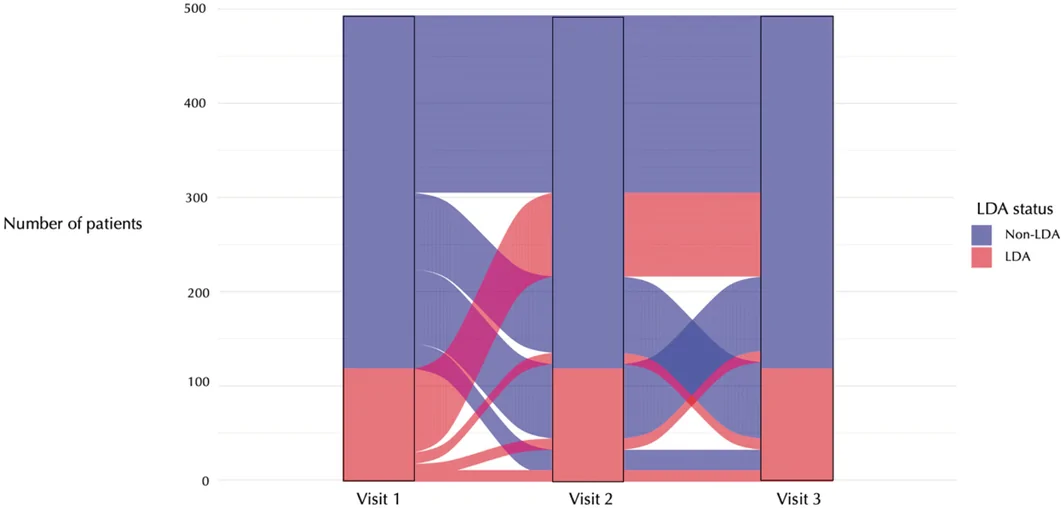
Alluvial plot of prospective stability of the LDA group in ATLANTIS. We assessed LDA at all 3 timepoints of ATLANTIS to assess how stable LDA was across the 52 weeks. Blue shading indicates patients who began the study not in LDA, whereas red shading indicates patients who began the study in LDA. The flows show where these patients moved to and from between each visit. LDA, low disease activity.

The LDA tool was evaluated to explore its associations with clinical outcomes, as shown in Figure 3a–d. At baseline, significantly more patients in LDA were from GINA steps 1–3 compared to classes 4–5 (*p* = 0.002), supporting the expected link with lower disease severity or duration (Figure 3c). After 52 weeks, patients identified by LDA at baseline were 4.4 times more likely to achieve 3C remission [OR 4.43 (2.80, 7.10)] and 3.5 times more likely to achieve 4C remission [OR 3.46 (2.23, 5.43)] (Figure 3d). Additionally, they were more than twice as likely to report improved quality of life [OR 2.07 (1.65, 2.60)] (Figure 3d). With respect to adverse outcomes, patients in LDA at baseline were 57% less likely to experience at least one future exacerbation [OR 0.43 (0.22, 0.85)] (Figure 3d). The prospective stability of LDA is depicted in Figure 4.

**FIGURE 5:**
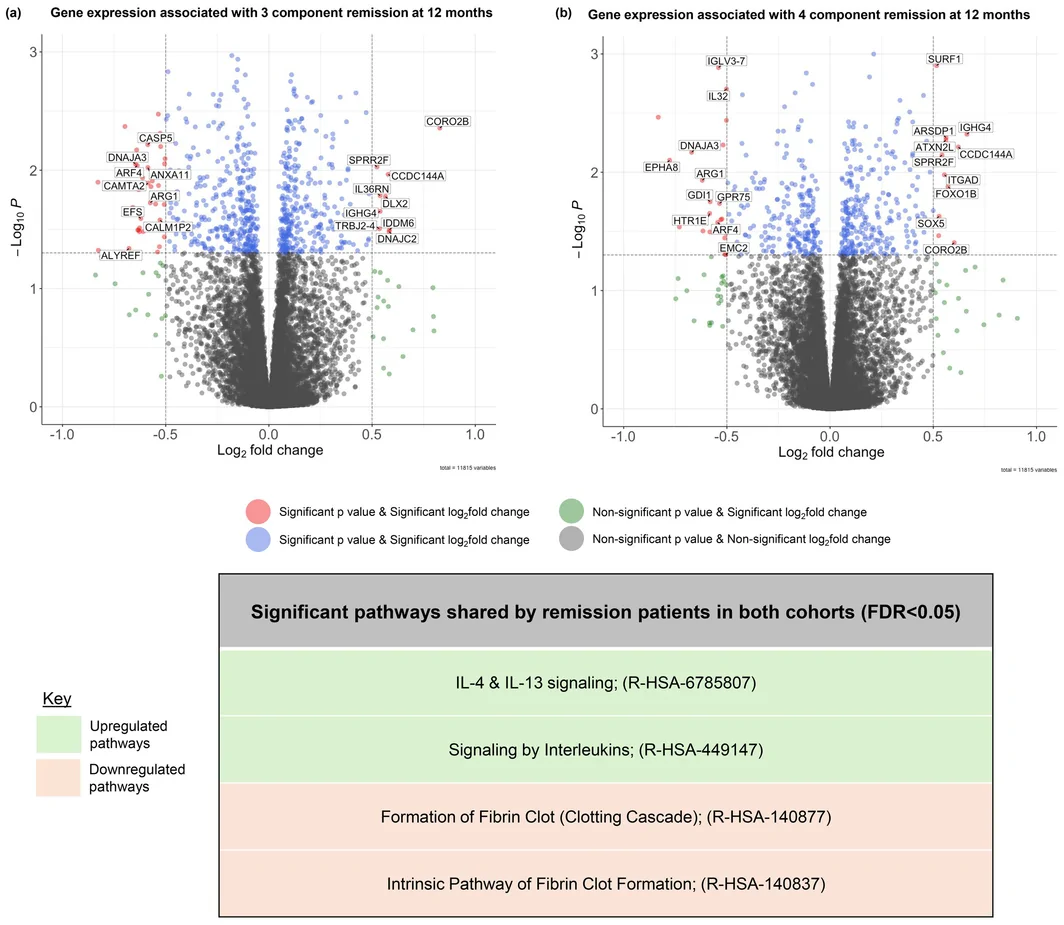
(a) Volcano plot of DEGs for 3‐component clinical remission in ATLANTIS (nasal brushings, *n* = 295). (b) Volcano plot for 4‐component remission. Unadjusted *p*‐values are shown; no genes remained significant after Benjamini‐Hochberg correction. Red: *p* < 0.05 and |log_2_FC| > 0.5; blue: *p* < 0.05; green: |log_2_FC| > 0.5. Horizontal and vertical dashed lines indicate significance thresholds. Key genes are labelled. (c) Shared significantly enriched Reactome pathways from DEGs common to both remission definitions, FDR‐corrected (*p* < 0.05). Pathways are colour‐coded by direction and annotated with Reactome IDs. DEG, differentially expressed gene; FDR, false discovery rate.

Principal components analysis (PCA) did not adequately capture the variation in gene expression related to remission status. Therefore, further gene expression analysis was conducted, with results visualised using volcano plots in Figure 5a,b. A total of 48 and 35 differentially expressed genes (DEGs) were identified for 3C and 4C remission, respectively, using a significance threshold of *p* < 0.05. However, after applying Benjamini‐Hochberg correction for multiple testing, no DEGs remained statistically significant.

Differential protein expression (DEP) analysis was performed on sputum proteomics data from the U‐BIOPRED subset in relation to 3C remission. This analysis identified 50 DEPs at *p* < 0.05, 7 DEPs at *p* < 0.01, but none remained significant after Benjamini‐Hochberg correction at either threshold (Table S4).

DEGs and DEPs in ATLANTIS and U‐BIOPRED respectively were subjected to pathway enrichment analysis (PEA) using the Reactome database. In individuals in remission, shared upregulated pathways included ‘Signalling by Interleukins’ and ‘Interleukin‐4 and Interleukin‐13 signalling’ (Figure 5c). Conversely, downregulated pathways included the ‘Intrinsic Pathway of Fibrin Clot Formation’ and ‘Formation of Fibrin Clot (Clotting Cascade)’ (Figure 5c). Pathway references can be found in the Supporting Information (supplementary methods section).

Two coagulation pathways (R‐HSA‐140877 and R‐HSA‐140837) were associated with the prolylcarboxypeptidase (PRCP) gene, which was measured in the nasal transcriptomics dataset of U‐BIOPRED (Figures 5c and S2). A correlation analysis was performed to assess the relationship between PRCP gene expression and LDA in the U‐BIOPRED cohort. PRCP expression was lower in the remission group, although the difference was not statistically significant (Wilcoxon *p* = 0.3). Nonetheless, within this group, PRCP expression showed a significant, albeit moderate, inverse correlation with LDA score (Spearman *ρ* = −0.51, *p* = 0.011) (Figure S2).

## 4 Discussion

This post hoc exploratory analysis of ATLANTIS revealed that clinical remission is related to small airways dysfunction and identified a potential low disease activity profile comprising ACQ‐6, FeNO, SADT, BEC, and pre‐bronchodilator FEV_1_ that was prospectively associated with fewer exacerbations, a higher likelihood of remission, and improved quality of life. This is the first study to demonstrate that small airways dysfunction is an independent risk factor for failure to achieve clinical remission. Additionally, through a combined analysis of nasal transcriptomics from ATLANTIS and sputum proteomics from U‐BIOPRED, we identified and validated associations between IL‐4Rα and coagulation pathways with increased and decreased remission likelihood, respectively. To our knowledge, this is the first report to identify and replicate biological pathways linked to asthma remission.

In ATLANTIS, 48% and 45% of patients achieved 3C and 4C remission respectively. In contrast, 3% and 1% of patients in the U‐BIOPRED cohort achieved 3C and 4C remission, respectively. This difference may be attributed to the U‐BIOPRED cohort comprising patients with severe asthma, whereas ATLANTIS included individuals across all disease severities. This highlights the impact of airway remodelling and the irreversible structural changes associated with more severe forms of asthma and therefore a lower likelihood of remission in this category of patients [23].

Subjects in remission at baseline exhibited distinct clinical characteristics consistent with prior reports [15, 17, 19]. Notably, and to our knowledge for the first time, we found that higher SADT, indicative of less SAD, was associated with remission. As expected, remission was also linked to lower BNC and Neu%, though, interestingly, BEC was also lower in remission patients, contrasting with earlier studies [15, 19]. When stratifying BEC by clinically accepted cut‐offs, most patients in remission fell within the low‐to‐moderate range. While not statistically significant, these patients also tended to have moderate‐to‐high FeNO levels. Despite lower BEC, the presence of elevated FeNO alongside reduced BNC and Neu% suggests ongoing T2 inflammation, which has previously been linked to remission [17, 19]. This may indicate that T2 inflammation contributes to remission, but that BEC alone may be an unreliable biomarker.

Regression models accompanied by ROC curve analysis demonstrated adequate discriminative performance, with model metrics indicating good diagnostic accuracy. Seven significant predictors of remission were identified, including the SADT score, highlighted for the first time. Interestingly, the physiological SAD score was not a significant predictor, suggesting that self‐reported small airways symptoms may be more predictive of remission than objective measures.

We subsequently developed a novel disease scoring tool to assess LDA. As anticipated in a non‐interventional study, approximately half of the participants never achieved LDA. Notably, LDA status demonstrated prospective instability, which may be attributable to the use of a proportional threshold (i.e., the lower quartile), inherently limiting LDA classification to 25% of participants at each visit and contributing to this observed variability. LDA was associated with mild‐to‐moderate asthma, supporting its validity in reflecting underlying disease severity. The tool also aligned well with key prospective clinical outcomes, where LDA was linked to increased remission rates, fewer prospective exacerbations, and improved QoL at 12 months follow up. However, we duly acknowledge that further external validation is required before this potential LDA profile can be considered a fully established predictive instrument. Given that severe or non‐biologically treated asthmatics are unlikely to achieve remission under current definitions, LDA may serve as a valuable alternative treatment target or a ‘stepping stone’ toward remission.

No significant DEGs or DEPs at the FDR level were found to be associated with clinical remission in either cohort. Two primary biological pathways were associated with remission. First, the upregulation of interleukin‐13 (IL‐13) and interleukin‐4 (IL‐4) in patients in remission supports the hypothesis that T2‐high asthma is associated with remission, consistent with previous findings [17, 19]. Since IL‐4/13 pathways are influenced by ICS, one might attribute clinical remission to this effect; however, an alternative explanation is that these pathways play a role in barrier repair and maintaining homeostasis [24]. Furthermore, participation in a clinical trial with regular visits is likely to improve adherence and consequent asthma control. Second, in patients in remission, we also observed notable downregulation of haemostatic pathways in both cohorts, particularly ‘Formation of Fibrin Clot (Clotting Cascade)’ [25] and ‘Intrinsic Pathway of Fibrin Clot Formation’ [26]. These findings are biologically plausible given the established role of prothrombotic and profibrotic activity in severe asthma, characterised by elevated tissue factor, increased clotting factor expression, and enhanced airway thrombogenesis [27, 28, 29]. Even individuals with mild asthma have demonstrated dysregulation in anticoagulant mechanisms, such as impaired Activated Protein C (APC) systems [30], suggesting that suppression of procoagulant activity may contribute to remission.

Theoretically, therapeutically modulating haemostatic pathways has shown promise. For example, murine models treated with APC have exhibited reduced inflammatory markers, while inhaled heparin has demonstrated some capacity to mitigate AHR [31, 32]. Moreover, airway remodelling has been linked to haemostatic processes, with upregulation of plasminogen activator inhibitor‐1 (PAI‐1) in murine models reducing mucus and collagen deposition in the airways [33].

Future directions can be outlined in three key areas. First, our novel finding linking SAD to asthma remission warrants further investigation and confirmation with prospective studies. The SADT questionnaire spans 63 domains [22], and analysing which domains participants in remission scored highly in could provide deeper insights into this relationship. SAD has been linked to more severe asthma, increased exacerbations, and poorer asthma control, even when spirometry appears normal [34].

Our LDA tool shows promising potential. By accounting for asthma heterogeneity through specific trait targeting, we observe meaningful correlations between LDA scores and clinical outcomes. Before clinical implementation, the tool should be refined using an unstandardised factor analysis [35]. If validated in large, demographically similar cohorts, a numerical threshold for LDA could be established. This would pave the way for a clinical trial comparing conventional asthma management with an approach guided by our LDA tool, aiming to maintain patients within a targeted LDA range. The arm yielding superior long‐term outcomes such as higher remission rates or fewer exacerbations could indicate a more effective management strategy. Furthermore, by retrospectively applying our remission models and LDA tool to data from T2 biologic trials, we aim to test our hypothesis that biologics play a key role in inducing remission.

We also identified several key pathways associated with remission. To better understand their relevance, the temporal dynamics of potential biomarkers such as interleukins and clotting factors should be evaluated through longitudinal monitoring with remission as the clinical endpoint. Should these findings be replicated, the implicated pathways could serve as diagnostic or therapeutic targets, potentially complementing biologic therapies and, to a lesser extent, corticosteroids and bronchodilators. Whether modification of the coagulation pathways could facilitate remission remains an intriguing question. However, these conclusions remain speculative and are not intended as recommendations for current asthma management practices. We also assessed the relationship between LDA and PRCP gene expression in U‐BIOPRED. Importantly, these findings also validated the ATLANTIS results in an independent cohort and highlight the value of using continuous indices such as the LDA score in omics‐based analyses. By moving beyond binary classifications, the continuous nature of LDA enables the discovery of more nuanced biological associations.

Key strengths of this study included the use of real‐world data, a large sample size and the inclusion of discovery and validation cohorts to evaluate the relationship between DEGs and DEPs and asthma remission. While Multiple Imputation by Chained Equations was considered to address missing data, we opted not to include synthetic data to preserve the validity of our findings. A wide range of variables was examined, supporting existing evidence, particularly the pivotal role of biologics in inducing remission [15]. Additionally, we identified several novel associations and generated new hypotheses to guide future research.

As this was a post hoc observational analysis, the associations we report between small airways dysfunction, the LDA profile, and clinical outcomes reflect naturally occurring variation within the cohort rather than the effects of targeted treatments. We fully acknowledge that unmeasured factors may influence both disease activity and remission but were not controlled for in this analysis. ATLANTIS was a cross‐sectional observational study, where background therapy was stable during the follow up period or changes were made at the discretion of the treating clinician. This may have influenced the results of the study. The associations do not at present imply that modifying any specific domain within the LDA tool (e.g., SADT score, FeNO, eosinophils) will result in remission. The LDA profile is presented as a hypothesis‐generating framework that warrants prospective validation in interventional studies.

Limitations were present throughout all stages of this exploratory study. First, defining remission remains a subject of debate. To address this, we conducted a comprehensive sensitivity analysis to justify our chosen definition and performed a subgroup analysis of individuals who did not meet the three‐component remission criteria in ATLANTIS. Among potential thresholds, ‘ACQ‐6 < 1.5’ emerged as the most stringent, excluding 61% of participants. While some concerns may persist, particularly given a recent Delphi consensus recommending ‘ACQ‐6 < 0.75’ to better distinguish remission from clinical response [36], our definition aligns with multiple previous studies [15, 19]. Another limitation relates to the interpretative scope of our findings. This was a post hoc study, not designed to elucidate the underlying biology of remission, which may involve unknown genetic or molecular confounders. Additionally, the inclusion of numerous variables in our models introduces the potential for overfitting and Type I errors. Nevertheless, the models demonstrated acceptable specificity, and given the exploratory nature of this analysis, this limitation is not a significant concern.

A key limitation of this study is the use of two inherently different populations. Since mild‐to‐moderate asthmatics were not followed longitudinally in U‐BIOPRED, omics replication analyses were restricted to individuals with severe asthma. This, combined with the absence of biologic treatments, likely reduced the probability of remission in the U‐BIOPRED cohort compared to the general asthma population [37]. Another limitation involves differences in omic sampling: nasal brushings transcriptomics in ATLANTIS versus sputum proteomics in U‐BIOPRED, which may not be perfectly comparable, although both capture key airway processes relevant to asthma. Due to the limited number of patients in remission in U‐BIOPRED, we were unable to conduct a DEP analysis for 4C remission, which would have strengthened our protein enrichment analysis. Therefore, we acknowledge that our observations are supportive but not definitive evidence of mechanistic relevance.

In summary, we demonstrated that remission occurred in approximately 50% of patients who were predominantly treated with either inhaled or oral anti‐asthmatic therapies. Several predictors of remission were identified, importantly for the first time, including a link with SAD. Our LDA tool correlated strongly with quality of life, fewer prior and prospective exacerbations, and an increased likelihood of remission. We also identified novel biological pathways potentially underlying remission. Future directions should focus on harmonising definitions of remission, longitudinal validation, optimising the LDA tool for clinical application and exploring therapeutic strategies that target these emerging pathways.

## Funding

The ATLANTIS study was funded by Chiesi Farmaceutici. This *post hoc* analysis was supported by the NIHR Imperial Biomedical Research Centre (BRC). The views expressed are those of the authors and not necessarily those of the NIHR or the Department of Health and Social Care.

## Consent

All subjects gave written informed consent prior to any study activities.

## Conflicts of Interest

A.K. reports no conflicts of interest. R.C. reports institutional grants awarded from Asthma + Lung UK, Chiesi, AstraZeneca, and GlaxoSmithKline; serving on advisory boards for AstraZeneca and Vitalograph; personal fees (talks and drafting educational material) from AstraZeneca, Chiesi, Thorasys, and Vitalograph; and support attending meetings from AstraZeneca, Chiesi, NIOX, Sanofi‐Regeneron, and Vitalograph. N.Z.‐K. reports no conflicts of interest. E.Q. reports no conflicts of interest. I.M.A. reports institutional grants from GSK, MRC, EPSRC, Sanofi, and Sysmex; consulting fees from GSK; lecture fees from AstraZeneca, GSK, and Sunvou; serving on advisory boards for Kinaset, Sanofi, and GSK; and is the head of the asthma section for EAACI. S.S. reports advisory board and/or speaker fees from Astra Zeneca, GSK, Chiesi, Sanofi, Medscape, Areteia therapeutics, and is on the scientific steering group for the ATLANTIS consortium. C.B. has received grants and consultancy fees from 4D Pharma, Areteia, AstraZeneca, Chiesi, Genentech, GlaxoSmithKline, Mologic, Novartis, Regeneron Pharmaceuticals, Roche, and Sanofi paid to his Institution. B.B. has received honoraria for advisory boards or lectures from AstraZeneca, Chiesi Farmaceutici, GSK, Lusofarma, Menarini Gropu, Sanofi. D.S. has received personal fees from Aerogen, AstraZeneca, Boehringer Ingelheim, Chiesi, Cipla, CSL Behring, EpiEndo Pharmaceuticals, Genentech, Glenmark Pharmaceuticals, Gossamer Bio, GSK, Kinaset Therapeutics, Menarini Group, Novartis, PULMATRiX, Sanofi, Synairgen, Teva Pharmaceuticals, Theravance Biopharma, and Verona Pharma, and has a leadership or fiduciary role in the Global Initiative for Chronic Obstructive Lung Disease (science committee). J.K. reports grants, personal fees and non‐financial support from AstraZeneca, Boehringer Ingelheim, GSK; grants and personal fees from Chiesi, Teva; non‐financial support from Mundi Pharma; personal fees from MSD, COVIS Pharma, ALK‐Abello; grants from Valneva outside the submitted work; and Janwillem Kocks holds < 5% shares of Lothar Medtec GmbH and 72.5% of shares in the General Practitioners Research Institute. K.F.R. has received payment or fees for lectures, presentations, speakers bureaus, manuscript writing, or educational events from AstraZeneca, Berlin‐Chemie, Boehringer Ingelheim, Chiesi, GSK, Novartis, Regeneron Pharmaceuticals, Roche, and Sanofi, and has received fees for advisory board participation from AstraZeneca, Boehringer Ingelheim, Regeneron Pharmaceuticals, and Sanofi. U.S.‐A. is an employee of Chiesi Farmaceutici, Parma, Italy. M.v.d.B. reports research grants paid to Institution from GlaxoSmithKline, Chiesi, Genentech, and Roche. M.K. reports research grants paid to her institution from Sanofi, Areteia, American Lung Association, and National Institutes of Health; personal fees (consulting and speaking) from Chiesi, Sanofi, GlaxoSmithKline, Genentech/Roche, AstraZeneca, Regeneron, and Kinaset, and royalties as a section editor and author for UptoDate. A.P. reports grants from Chiesi, AstraZeneca, GlaxoSmithKline, and Sanofi; consultancy fees from Chiesi, AstraZeneca, GlaxoSmithKline, Sanofi, Avillion, Moderna, Roche, Regeneron, Zambon, Zentiva, and payment or honoraria for lectures, presentations, speakers' bureaus from Chiesi, AstraZeneca, GlaxoSmithKline, Zambon, Sanofi, Avillion, Regeneron, Moderna, Roche.

## Supporting information


**Data S1:** all70264‐sup‐0001‐Supinfo.docx.

## Data Availability

The data that support the findings of this study are available from the corresponding author upon reasonable request.
