# Supplementary material for: Small Airways Dysfunction and Remission in Adults With Asthma: A Longitudinal Exploratory Analysis of the AssessmenT of smalL Airways involvemeNT In aSthma (ATLANTIS) Study
Source: Allergy. 2026 Feb 18;81(9):3185–96. doi: 10.1111/all.70264 (PMC13569693; doi:10.1111/all.70264)
Supplement: Supplementary file 1 [file ALL-81-3185-s001.docx]

Title:	Small airways dysfunction and remission in adults with asthma: A post hoc longitudinal analysis of the ATLANTIS study

Authors:	Akshi Kumar^1^*

Rory Chan PhD, FRCPE^2^*

Nazanin Zounemat-Kermani, MSc, PhD^1,3^

Ellie Quek, MBChB^1^

Ian M. Adcock, PhD^1^

Christopher Brightling, PhD FMedSci^4^

Dave Singh, MD^5^

Janwillem Kocks, MD, PhD^6^

Leonardo Fabbri, MD^7^

Alberto Papi, MD^7^

Klaus Rabe, MD, PhD, FERS^8^

Marielle van der Deijl^9^

Maarten van den Berge, MD, PhD^10^

Monica Kraft, MD^11^

Salman Siddiqui, PhD, FRCP^1^

*Contributed equally as co-first authors

Affiliation:	1. National Heart and Lung Institute, NIHR Imperial Biomedical Research Centre, Imperial College London, London, UK

2. University of Dundee, School of Medicine, Dundee, UK

3. Data Science Institute, Imperial College London, London, UK

4. Institute for Lung Health, National Institute for Health Research Biomedical Research Centre, University of Leicester, Leicester, UK

5. Centre for Respiratory Medicine and Allergy, University of Manchester, Manchester University NHS Foundation Trust, Manchester, UK

6. General Practitioners Research Institute, Groningen, The Netherlands

7. Department of Translational Medicine, University of Ferrara, Ferrara, Italy

8. LungenClinic Grosshansdorf, Grosshansdorf, Germany; Airway Research Center North, German Center for Lung Research, Grosshansdorf, Germany; Christian-Albrechts University of Kiel, Kiel, Germany

9. Global Medical Affairs, Chiesi Farmaceutici S.p.A. Parma, Italy

10. Department of Pulmonary Diseases, University of Groningen, University Medical Center Groningen, Groningen, The Netherlands

11. Department of Medicine, Icahn School of Medicine at Mount Sinai, Mount Sinai Health System, New York, NY USA

Corresponding author:	Professor Salman Siddiqui

National Heart and Lung Institute, NIHR Imperial Biomedical Research Centre, Imperial College London, London, UK

E-mail: s.siddiqui@imperial.ac.uk

Tel: +44 (0)20 7594 5303

Conflicts of Interest:

AK reports no conflicts of interest.

RC reports institutional grants awarded from Asthma + Lung UK, Chiesi, AstraZeneca and GlaxoSmithKline; serving on advisory boards for AstraZeneca and Vitalograph; personal fees (talks and drafting educational material) from AstraZeneca, Chiesi, Thorasys and Vitalograph; and support attending meetings from AstraZeneca, Chiesi, NIOX, Sanofi-Regeneron and Vitalograph.

SS reports advisory board and/or speaker fees from Astra Zeneca, GSK, Chiesi, Sanofi, Medscape, Areteia therapeutics and is on the scientific steering group for the ATLANTIS consortium

IA reports institutional grants from GSK, MRC, EPSRC, Sanofi and Sysmex; consulting fees from GSK; lecture fees from AstraZeneca, GSK and Sunvou; serving on advisory boards for Kinaset, Sanofi and GSK; and is the head of the asthma section for EAACI.

CB has received grants and consultancy fees from 4D Pharma, Areteia, AstraZeneca, Chiesi, Genentech, GlaxoSmithKline, Mologic, Novartis, Regeneron Pharmaceuticals, Roche and Sanofi paid to his Institution.

DS has received personal fees from Aerogen, AstraZeneca, Boehringer Ingelheim, Chiesi, Cipla, CSL Behring, EpiEndo Pharmaceuticals, Genentech, Glenmark Pharmaceuticals, Gossamer Bio, GSK, Kinaset Therapeutics, Menarini Group, Novartis, PULMATRiX, Sanofi, Synairgen, Teva Pharmaceuticals, Theravance Biopharma, and Verona Pharma, and has a leadership or fiduciary role in the Global Initiative for Chronic Obstructive Lung Disease (science committee).

JK reports grants, personal fees and non-financial support from AstraZeneca, Boehringer Ingelheim, GSK; grants and personal fees from Chiesi, Teva; non-financial support from Mundi Pharma; personal fees from MSD, COVIS Pharma, ALK-Abello; grants from Valneva outside the submitted work; and Janwillem Kocks holds <5% shares of Lothar Medtec GmbH and 72.5% of shares in the General Practitioners Research Institute.

LF has received honoraria for advisory boards or lectures from Alfasigma, AstraZeneca, Chiesi Farmaceutici, GSK, Lusofarma, Novartis, and Verona Pharma.

KFR has received payment or fees for lectures, presentations, speakers bureaus, manuscript writing, or educational events from AstraZeneca, Berlin-Chemie, Boehringer Ingelheim, Chiesi, GSK, Novartis, Regeneron Pharmaceuticals, Roche, and Sanofi, and has received fees for advisory board participation from AstraZeneca, Boehringer Ingelheim, Regeneron Pharmaceuticals, and Sanofi.

MvdB reports research grants paid to Institution from GlaxoSmithKline, Chiesi, Genentech, and Roche.

MK reports research grants paid to her institution from Sanofi, Areteia, American Lung Association and National Institutes of Health; personal fees (consulting and speaking) from Chiesi, Sanofi, GlaxoSmithKline, Genentech/Roche, AstraZeneca, Regeneron and Kinaset, and royalties as a section editor and author for UptoDate.

AP reports grants from Chiesi, AstraZeneca, GlaxoSmithKline and Sanofi; consultancy fees from Chiesi, AstraZeneca, GlaxoSmithKline, Sanofi, Avillion, Moderna, Roche, Regeneron, Zambon, Zentiva and payment or honoraria for lectures, presentations, speakers’ bureaus, from Chiesi, AstraZeneca, GlaxoSmithKline, Zambon, Sanofi, Avillion, Regeneron, Moderna, Roche.

Word count: 3,644

**Tables:** 1

**Figures:** 5

**Key Words:** Asthma, Disease Exacerbation, Spontaneous Remission, Remission Induction, Gene Expression Analysis, Factor Analysis

**Funding:** The ATLANTIS study was funded by Chiesi. This *post hoc* analysis was supported by the NIHR Imperial Biomedical Research Centre (BRC). The views expressed are those of the authors and not necessarily those of the NIHR or the Department of Health and Social Care.

**Contents**

Page 5	U-BIOPRED author list

Page 9	Definitions table

Page 11	Supplementary methods

Page 14	Supplementary Table 1: Factor analysis results from ATLANTIS and U-BIOPRED

Page 15	Supplementary Table 2: List of significant genes from Differential Gene Expression of 3-component remission in ATLANTIS

Page 16	Supplementary Table 3: List of significant genes from Differential Gene Expression of 4-component remission in ATLANTIS

Page 17	Supplementary Table 4: List of significant genes from Differential Gene Expression of 3-component remission in U-BIOPRED

Page 18	Supplementary Tables S5: List of significantly upregulated pathways

Page 19	Supplementary Tables S6: List of significantly downregulated pathways

Page 20	Supplementary Tables S7: List of significantly upregulated and downregulated pathways

Page 21	Supplementary Figure 1. U-BIOPRED Consort diagram

Page 22	Supplementary Figure 2. Correlation between PRCP gene expression and Low Disease Activity groups in the U-BIOPRED cohort

Page 23	Supplementary Tables S8

Page 24	References

**U-BIOPRED Author list**

| **Name** | **Email** | **Institution** |
| --- | --- | --- |
| Adam Platt | [Adam.Platt@astrazeneca.com](mailto:Adam.Platt@astrazeneca.com) | AZ |
| Adnan Azim | [adnan.azim@astrazeneca.com](mailto:adnan.azim@astrazeneca.com) | AZ |
| Ali Versi | [ali.versi@imperial.ac.uk](mailto:ali.versi@imperial.ac.uk) | Imperial College |
| Alvar Agusti | aagusti@clinic.cat | Hospital clinic de Barcelona |
| Amanda Roberts | amandaroberts@ntlworld.com |  |
| Ana Marie Bourke | advocacy@asthmasociety.ie | Asthma Society of Ireland |
| Ana Sousa | ana.x.sousa@gsk.com | GSK |
| Andrew Bush | a.bush@imperial.ac.uk |  |
| Andy Wagener | A.H.Wagener@amc.uva.nl |  |
| Anke-Hilse Maitland van der Zee | a.h.maitland@amsterdamumc.nl | Amsterdal UMC |
| Anna James | Anna.James@ki.se | Karolinska Institutet |
| Anna-Carin Olin | anna-carin.olin@amm.gu.se |  |
| Antonelli Raffaele | r.antonelli@unicampus.it |  |
| Ariane Wagener | a.wagener@amsterdamumc.nl | AMC Amsterdam |
| Aruna Bansal | aruna.t.bansal@acclarogen.com | Acclarogen |
| Barbro Dahlén | barbro.dahlen@ki.se |  |
| Bel E.H.D. | e.h.bel@amsterdamumc.nl |  |
| Bertrand | bdemeulder@eisbm.org |  |
| Björn Nordlund | [bjorn.nordlund@ki.se](mailto:bjorn.nordlund@ki.se) | Karolinska Institutet |
| Braun Armin | armin.braun@item.fraunhofer.de |  |
| Brinkman P. | p.brinkman@amsterdamumc.nl |  |
| Charles Auffray | cauffray@eisbm.org |  |
| Chris Compton | chris.h.compton@gsk.com |  |
| Cilla Soderhall | [cilla.soderhall@ki.se](mailto:cilla.soderhall@ki.se) | Karolinska Institutet |
| Clare Murray | clare.murray@manchester.ac.uk | University of Manchester |
| Courtney Coleman | courtney.coleman@europeanlung.org | European Lung Foundation |
| Craig Wheelock | [craig.wheelock@ki.se](mailto:craig.wheelock@ki.se) | Karolinska Institutet |
| Cunoosamy Danen /US | danen.cunoosamy@sanofi.com | Sanofi |
| David Supple | davidsupple@gmail.com |  |
| Deborah Peñate Gómez | dgomez@auk-blf.org.uk |  |
| Dieter Maier | dieter.maier@biomax.com | Biomax Informatics AG |
| Dominick Shaw | dominic.shaw@nottingham.ac.uk | Now at Leicester (des21@leicester.ac.uk) |
| Dorothy de Sousa | dorothy.de_sousa@boehringer-ingelheim.com |  |
| Erik Golbach | e.m.golbach@amsterdamumc.nl | Amsterdam UMC |
| Ewa Nizankowska | [ewan@ghml.pl](mailto:ewan@ghml.pl) | Krakow |
| Ewa Wikström Jonsson | [eva.wikstrom-jonsson@ki.se](mailto:eva.wikstrom-jonsson@ki.se) | Karolinska Institutet |
| Fan Chung | f.chung@imperial.ac.uk | Imperial |
| FANER ROSA (CIBER) | rfaner@clinic.cat | CIBERES |
| Fleming Louise J | l.fleming@imperial.ac.uk |  |
| Florian Singer | [florian.singer@uzh.ch](mailto:florian.singer@uzh.ch) |  |
| Fred Baribaud | [Frederic.Baribaud@bms.com](mailto:Frederic.Baribaud@bms.com) |  |
| Freda Yang | [freda.yang@imperial.ac.uk](mailto:freda.yang@imperial.ac.uk) | Imperial College |
| Graham Roberts | g.c.roberts@soton.ac.uk |  |
| Gautam Damera | gautam.damera@tevapharm.com |  |
| Graham Clarke | [graham.clarke1@astrazeneca.com](mailto:graham.clarke1@astrazeneca.com) | AZ |
| Gunilla Hedlin | gunilla.hedlin@ki.se | Karolinska Institutet |
| Hans Michael Haitchi | H.M.Haitchi@soton.ac.uk |  |
| Hashimoto S. (Simone) | s.hashimoto@amsterdamumc.nl | Amsterdam UMC |
| Heribert.Staudinger@sanofi.com | heribert.staudinger@sanofi.com |  |
| Hitasha Rupani | h.rupani@nhs.net |  |
| Huanyu Zhou | huanyu.zhou01@tevapharm.com |  |
| Ian M Adcock | ian.adcock@imperial.ac.uk | Imperial College London |
| Ibrahim M.I.A. (Mahmoud) | m.i.ibrahim@amsterdamumc.nl | University of Amsterdam |
| Ildiko Horvath | [ildiko.horvath@koranyi.hu](mailto:ildiko.horvath@koranyi.hu) | Budapest |
| Jacek Musial | jacek.musial@uj.edu.pl | Krakow |
| Jaumont Xavier | xavier.jaumont@novartis.com |  |
| Jennifer Kearley | jennifer.kearley@astrazeneca.com | Astra Zeneca |
| Jenny Mjösberg | [jenny.mjosberg@ki.se](mailto:jenny.mjosberg@ki.se) | Karolinska Institutet |
| Jens Hohlfeld | jens.hohlfeld@item.fraunhofer.de |  |
| Johan Kolmert | johan.kolmert@ki.se | Karolinska Institutet |
| Jon Konradsen | [jon.roald.konradsen@ki.se](mailto:jon.roald.konradsen@ki.se) | Karolinska Institutet |
| Joost Brandsma | J.Brandsma@soton.ac.uk | Henry M. Jackson Foundation |
| Jorgen Ostling | Jorgen.Ostling@pexa.se | PExA |
| Jorgen Vestbo | jorgen.vestbo@manchester.ac.uk |  |
| Julian Nehrig | [julian.nehrig@item.fraunhofer.de](mailto:julian.nehrig@item.fraunhofer.de) | Fraunhofer ITEM Hannover |
| Julie Corfield | juliecorfield@areteva.com | Areteva |
| Kai Sun | k.sun@imperial.ac.uk | Imperial |
| Karen Affleck | karen.x.affleck@gsk.com | GSK |
| Karen Needham | auntykaz23@icloud.com | ERS PAG |
| Khokhlovich Edward | edward.khokhlovich@novartis.com | Novartis Institutes for Biomedical Research |
| Kissné Dr. Horváth Ildikó | ildiko.horvath@koranyi.hu |  |
| Kjell Alving | kjell.alving@kbh.uu.se | Uppsala University |
| KOTS Maxim | m.kots@chiesi.com |  |
| Krisnah Poinasamy | kpoinasamy@auk-blf.org.uk | Asthma UK & British Lung Foundation |
| Kristoffer Ostridge | [kristoffer.ostridge@astrazeneca.com](mailto:kristoffer.ostridge@astrazeneca.com) | AZ |
| Lars Andersson | lars.i.andersson@ki.se | Karolinska Institutet |
| Liam Heaney | l.heaney@qub.ac.uk | Queens University Belfast |
| Liling Warren | liling.warren02@tevapharm.com | Teva |
| Lottie Renwick | crenwick@auk-blf.org.uk | Asthma UK & British Lung Foundation |
| Loza Matthew [JRDUS] | mloza@its.jnj.com |  |
| Maria Sparreman Mikus | [sparreman.mikus@gmail.com](mailto:sparreman.mikus@gmail.com) | Karolinska Institutet |
| Martijn Spruit | martijnspruit@ciro-horn.nl | CIRO/ UM |
| Massimo Caruso | mascaru@unict.it | University of Catania |
| Maurik van den Heuvel | maurik.vandenheuvel@breathomix.com |  |
| Mirco Govoni | m.govoni@chiesi.com | Chiesi |
| Monika Ezerskyte | [monika.ezerskyte@ki.se](mailto:monika.ezerskyte@ki.se) |  |
| Montuschi Paolo | paolo.montuschi@unicatt.it | Catholic University of the Sacred Heart, Rome, Italy |
| Norbert Krug | norbert.krug@item.fraunhofer.de |  |
| Oksana Tenselius | [oksana.tenselius@ki.se](mailto:oksana.tenselius@ki.se) | Karolinska Institutet |
| Olsson Henric K | henric.k.olsson@astrazeneca.com |  |
| P.W. Hekking | p.w.hekking@amc.uva.nl |  |
| Pascal Chanez | pascal.chanez@univ-amu.fr |  |
| Pennazza Giorgio | g.pennazza@unicampus.it | UCBM |
| Per Bakke | Per.Bakke@uib.no |  |
| Peter Howarth | [peter.h.howarth@gsk.com](mailto:peter.h.howarth@gsk.com) |  |
| Pippa Powell | Pippa.Powell@europeanlung.org |  |
| Rao Navin [JRDUS] | nrao2@its.jnj.com |  |
| Ratko Djukanovic | r.djukanovic@soton.ac.uk | University of Southampton |
| Renaud Louis | r.louis@chu.ulg.ac.be |  |
| Rianne de Vries | rianne.devries@breathomix.com |  |
| Richard Knowles | prof.rgknowles@btinternet.com | Knowles Consulting (retired) |
| Ruihua Hou | r.hou@soton.ac.uk |  |
| Sabina Škrgat | sabina.skrgat@kclj.si | University Clinical Centre Ljubljana |
| SALAS MARTA (ICR) | masalas@clinic.cat |  |
| Sam Alf.Sigmund | ralf.sigmund@boehringer-ingelheim.com |  |
| Sam Usanna.Palkonen | susanna.palkonen@efanet.org | EFA |
| Sam Walker | swalker@asthma.org.uk |  |
| Santonico Marco | m.santonico@unicampus.it |  |
| Saqi Mansoor | mansoor.saqi@imperial.ac.uk |  |
| Sara Barsch Cornacchini | [sara.cornacchini@ki.se](mailto:sara.cornacchini@ki.se) | Karolinska Institutet |
| Schofield J.P. | [james.schofield@topmd.co.uk](mailto:james.schofield@topmd.co.uk) |  |
| Sharon Mumby | [s.mumby@imperial.ac.uk](mailto:s.mumby@imperial.ac.uk) | Imperial College |
| Sigrid DaVeiga | Sigrid.daveiga@tevapharm.com | Teva |
| Skipp P.J. | pjss@soton.ac.uk | University of Southampton |
| Sofia Romagosa | sofia.romagosa@efanet.org | EFA |
| Stephen Fowler | stephen.fowler@manchester.ac.uk |  |
| Sterk P.J. (Peter) | [petersterk25@gmail.com](mailto:petersterk25@gmail.com) | Amsterdam UMC |
| Susan Wilson | S.J.Wilson@soton.ac.uk | University of Southampton (retired) |
| Sven-Erik Dahlén | sven-erik.dahlen@ki.se | Karolinska Institutet |
| Thomas Geiser | Thomas.Geiser@insel.ch |  |
| Thomas Sandström | thomas.sandstrom@umu.se | Umeå University |
| Tim Higenbottam | tim-higenbottam1@live.co.uk |  |
| Tony Postle | A.D.Postle@soton.ac.uk |  |
| Uddin Mohib | mohib.uddin@astrazeneca.com | AstraZeneca |
| Urs Frey | Urs.Frey@ukbb.ch |  |
| Valentyna Yasinska | [valentyna.yasinska@ki.se](mailto:valentyna.yasinska@ki.se) | Karolinska Institutet |
| Yi-Ke Guo | y.guo@imperial.ac.uk |  |
| Yusef Badi | [yusefbadi@gmail.com](mailto:yusefbadi@gmail.com) | Imperial College |
| Zorica Lazic | zoricalazickg@gmail.com | Clinical Center Kragujevac, clinic for pulmonary disease |
| Zounemat Kermani Nazanin | n.zounemat-kermani13@imperial.ac.uk |  |
| Zsuzsanna Csoma | csoma@koranyi.hu | National Koranyi Institute for Pulmonology |
| Åsa Wheelock | asa.wheelock@ki.se | Karolinska Institutet |

**Definitions table**

| **Low disease activity (LDA)** | Low disease activity (LDA) in this study refers to a composite clinical state derived through factor analysis of five variables including ACQ-6, FeNO, SADT score, blood eosinophil count, and pre-bronchodilator FEV₁, that together quantify the reversible components of asthma disease burden. Lower composite scores indicate lower disease activity, and patients in the lowest quartile were classified as being in LDA. This state reflects milder, more stable disease and was strongly associated with higher remission likelihood, improved quality of life, and reduced future exacerbation risk. |
| --- | --- |
| **Small airways dysfunction (SAD)^1^** | Small airways dysfunction (SAD) refers to impaired function of the peripheral airways, typically those less than 2 mm in internal diameter, which are a major site of airflow limitation in asthma. SAD arises from inflammation, airway wall thickening, mucus plugging, and ventilation heterogeneity within these distal airways. SAD is often not detected by routine spirometry but is strongly associated with poor symptom control, increased exacerbation frequency and more severe disease. |
| **Small airways dysfunction questionnaire tool (SADT)^2^** | The Small Airways Dysfunction Tool (SADT) is a validated patient-reported questionnaire designed to detect symptoms and clinical features associated with small airways dysfunction in asthma. It aggregates responses across multiple domains such as exertional breathlessness, wheeze, nocturnal symptoms, and environmental triggers to generate a summary score, with higher scores indicating fewer small airway-related symptoms. It is intended to support identification of small airways dysfunction as a treatable trait in both research and clinical practice. |
| **Small airways dysfunction physiological score^3^** | The small airways dysfunction physiological score is an objective, composite measure derived from multiple lung function tests such as oscillometry, spirometry indices sensitive to distal airway narrowing, and inert gas washout, to quantify physiological abnormalities in the small airways. Higher scores indicate greater impairment of peripheral airway function. Unlike the SADT questionnaire, which captures symptoms, the SAD score reflects measurable physiological dysfunction within the distal bronchial tree. |

**Supplementary methods**

The Asthma Control Questionnaire (ACQ) is a tool used to assess symptom control over the past week. Scores range from 0 (indicating optimal control) to 6. Scores above 1.5 indicate severe, uncontrolled asthma. The Asthma Quality of Life Questionnaire (AQLQ) is a questionnaire designed to evaluate the physical and emotional impact of asthma. Scores range from 1 (severely impaired) to 7 (no impairment), with a minimal clinical important difference of 0.5. The Small Airways Dysfunction (SAD) Physiological Score is a scoring system for SAD based on physiological measures such as impulse oscillometry and body plethysmography.^3^ Higher scores reflect more severe SAD.

The Small Airways Dysfunction questionnaire Tool (SADT) is a patient-reported questionnaire developed to identify individuals with symptoms suggestive of small airways involvement in asthma.^2^ Its development was grounded in qualitative research, including patient interviews, focus groups, and expert clinical review, to ensure that the items reflected symptom patterns attributable to dysfunction of the distal airways. The final instrument comprises 63 items covering a broad range of domains, such as exertional breathlessness, wheeze, nocturnal symptoms, environmental and weather-related triggers, upper-airway symptoms, and activity limitation. Each item is scored on a three-point scale (“No,” “Yes,” “Unknown”), with higher total scores indicating fewer symptoms consistent with small airways dysfunction. Although designed as a screening tool rather than a diagnostic instrument, the SADT has demonstrated good internal consistency and construct validity in asthma populations.

Within the ATLANTIS programme, the SADT was administered at baseline and follow-up visits, and responses were evaluated against a physiologically derived small airways dysfunction (SAD) classification. This physiological classification was generated from a composite score based on a structural equation model incorporating multiple tests of small airway function, including spirometry indices, oscillometry measures, and lung volume assessments. Logistic regression models were then developed to determine whether SADT responses, alone or in combination with readily available clinical data, could predict physiologically defined SAD. Three models were evaluated: a “Bronze” model incorporating patient characteristics and a highly informative SADT item; a “Silver” model that added spirometry (FEV₁% predicted); and a “Gold” model that additionally incorporated oscillometry. Across these models, one questionnaire item, “I sometimes wheeze when I am sitting or lying quietly”, showed the strongest and most consistent association with physiological SAD.

Model performance improved progressively with the addition of physiological measurements. The Bronze model demonstrated moderate discrimination (AUC 0.74), the Silver model showed substantially improved accuracy with spirometry included (AUC 0.87), and the Gold model incorporating oscillometry achieved excellent performance (AUC 0.96). These findings indicate that SADT can function as a practical screening tool for small airways dysfunction, particularly in settings where physiological assessments are limited, and can be further strengthened when paired with basic lung-function data. Although a minimum clinically important difference has not yet been formally established, the SADT provides a feasible and scalable approach to identifying probable small airways involvement in clinical and research settings.

GINA classifications and type 2 biomarkers were categorised using clinically established cut-offs (GINA: 1, 2–3, 4–5; BEC: <15, 0.15–0.30, ≥0.30×10⁹ cells/L; FeNO: <25, 25–50, ≥50 ppb), with the lowest category serving as the reference group in regression models.

For the low disease activity (LDA) analysis, patients with complete baseline data for all tested variables were included in the baseline analysis, while a more limited cohort with complete follow-up data for all variables was used for the longitudinal analysis. The full datasets were accessed via tranSMART, an open-source translational medicine data platform,^4^ and were supported by the European Translational Information and Knowledge Management Services (eTRIKS) project.

Baseline characteristics were analysed using the Mann-Whitney U test, Chi-squared test, or Fisher’s exact test, depending on the distribution and type of data. Multivariate logistic regression models were built using variables that showed statistical significance. Model performance was evaluated using Receiver Operating Characteristic (ROC) curves, area under the curve (AUC), and the Akaike Information Criterion (AIC). To assess LDA, an exploratory standardised factor analysis was conducted on five variables, ACQ-6, FeNO, SADT, blood eosinophil count (BEC), and pre-bronchodilator FEV_1_, to generate disease activity scores (**Supplementary Table S1**). Lower scores indicated lower disease activity, with the lowest quartile classified as LDA. LDA status at baseline was then compared with both baseline and longitudinal clinical outcomes. A significance threshold of p<0.05 was applied for analyses related to remission and LDA.

All statistical analyses were conducted through R Statistical Software, Version 2023 12.0+369 (Posit Software, PBC, Boston, MA.).^5^ Differential Gene Expression was conducted with DESeq2 (1.42.1) with a negative binomial generalised linear model.^6^ Differential gene expression analysis was conducted using the limma package (v3), applying linear models with empirical Bayes moderation. Factor analysis was conducted with Psych (2.4.3) with Ordinary Least Squares method to generate minimum residual solution.^7^ A full list of R packages can be accessed in Supplementary Materials. Pathway enrichment analysis was conducted through Reactome databases, 2022 by Posit Software, PBC.^8^

In ATLANTIS, total RNA from nasal epithelium was extracted using the MiRNeasy kit from Qiagen, according to the manufacturers’ instructions. Ribosomal RNA was depleted using the rRNA depletion kit (New England Biolabs, NEB#E6310). Libraries were barcoded with unique molecular identifiers (UMI) to account for duplicate counts. A pool with concentration of 1.1nM of RNA-seq libraries was used for paired-end sequencing (2×150bp) with Novaseq 6000 sequencer (Illumina, San Diego, California, USA). Raw reads quality control was performed using FastQC. Reads were mapped to the human reference genome GRCh38 with the STAR aligner^9^ using gene annotation from Ensembl release 101 (ftp://ftp.ensembl.org). After alignment, UMI deduplication was done by filtering out reads wit duplicate UMI barcodes and identical chromosomal locations. We checked concordance between reported gender and sex-chromosome-associated expression. rRNA depletion and library preparation qualities were evaluated by calculating the proportion of uniquely mapped reads. The ratio of uniquely mapped reads (prior to UMI deduplication step) to the total number of reads was used to assess the library quality; five outlier samples with extremely low ratios (less than 12.5%) were excluded from further analysis.

In ATLANTIS, sputum was induced by inhalation of hypertonic saline solution and sputum plugs were collected from which sputum cells and sputum supernatants were obtained. Cell pellets were stored in RNA stabilization buffer (Norgen Biotek, Thorhold, Canada). RNA purity (RIN >6) was measured by Agilent Bioanalyser (Agilent, Santa Clara, Calif). Expression profiling was studied using Affymetrix U133 Plus 2.0 microarrays (Affymetrix, Santa Clara, Calif). Raw data were quality assessed and pre-processed by robust multi-array average normalization. Probes of low expression were filtered by robust multi-array signal analysis for values <5 and also for batch/technical effects. The intensity of the raw probe sets were log base 2 transformed and normalized by the robust multi-array average (RMA) method.

Principal component analysis (PCA) did not adequately explain the variation in gene expression related to remission status in the ATLANTIS cohort. As a result, differential gene expression analysis was conducted and visualised using volcano plots and heatmaps. This analysis identified 48 and 35 differentially expressed genes (DEGs) for 3C and 4C remission, respectively, using a significance threshold of p<0.05 (**Supplementary Tables S2 and S3**). However, none of these DEGs remained significant following Benjamini-Hochberg correction.

To address this, we applied a more stringent significance threshold of p<0.01, identifying 16 and 13 DEGs associated with 3C and 4C remission, respectively (**Supplementary Tables S2 and S3**). At the p<0.05 level, we found 5 commonly upregulated and 21 commonly downregulated DEGs (**Supplementary Tables S2 and S3**). Under the stricter p<0.01 threshold, only SPRR2F remained upregulated, while RBM8A, DNAJA3, SPEG, and IL32 were downregulated. However, no DEGs remained significant after Benjamini-Hochberg correction.

In the U-BIOPRED subset, sputum proteomics were analysed for differential protein expression (DEP) in relation to 3C remission. This analysis yielded 50 DEPs at p<0.05 and 7 DEPs at p<0.01, with none remaining significant after Benjamini-Hochberg correction at either threshold (**Supplementary Table S4**).

DEGs and DEPs in ATLANTIS and U-BIOPRED were subjected to pathway enrichment analysis (PEA) using the Reactome database. In individuals in remission, shared upregulated pathways included 'Signaling by Interleukins'^10^ and 'Interleukin-4 and Interleukin-13 signaling'^11^ (**Figure 5c**). Conversely, downregulated pathways included the 'Intrinsic Pathway of Fibrin Clot Formation'^12^ and 'Formation of Fibrin Clot (Clotting Cascade)'^13^

**Supplementary Tables S1. Factor analysis results from ATLANTIS and U-BIOPRED**

To generate our ‘disease activity’ scores, we ran a standardized exploratory factor analysis on 5 treatable traits, ACQ-6, SADT, BEC, Pre-BD FEV_1_ and FeNO. These generated 2 latent variables (MR1 and MR2) which explained variation patterns in our datasets of the 5 traits within the data – the MR weighting of each latent variable was applied to the factor loadings, to generate a single composite ‘disease activity’ score per patient.

The *psych* package was to conduct the FA in R.

(a) refers to the factor analysis in our ATLANTIS subset; (b) refers to the factor analysis in our U-BIOPRED subset

*MR: Minimum Residual; ACQ-6: Asthma Control Questionnaire-6; SADT: Small Airways Dysfunction Tool; BEC: Blood Eosinophil Count; Pre-BD FEV_1_: Pre Bronchodilator Fractional exhaled Volume in 1 second; FeNO: Fractional Exhaled Nitric Oxide*

**Supplementary Table S2. List of significant genes from Differential Gene Expression in nasal brushings of 3-component remission in ATLANTIS**

After conducting our differential gene expression on 3-component remission, we extracted significant genes on two main thresholds: log_2_(FC) > 0.5 or < -0.5; unadjusted p-value <0.05. We present the genes with log_2_(FC), unadjusted p-values and adjusted p-values with Benjamini-Hochberg correction. Genes are also marked if they also appear in the Differential Gene Expression of ATLANTIS with 4-component remission.

*ROC: Reciever Operating Characteristics; AUROC: Area Under Receiver Operating Characteristics curve; PPV: Positive Predictive Value; NPV: Negative Predictive Value; F1: F1 score*

**Supplementary Table S3. List of significant genes from Differential Gene Expression in nasal brushings of 4-component remission in ATLANTIS**

After conducting our differential gene expression on 4-component remission, we extracted significant genes on two main thresholds: log_2_(FC) > 0.5 or < -0.5; unadjusted p-value <0.05. We present the genes with log_2_(FC), unadjusted p-values and adjusted p-values with Benjamini-Hochberg correction. Genes are also marked if they also appear in the Differential Gene Expression of ATLANTIS with 3-component remission.

*ROC: Reciever Operating Characteristics; AUROC: Area Under Receiver Operating Characteristics curve; PPV: Positive Predictive Value; NPV: Negative Predictive Value; F1: F1 score*

**Supplementary Table S4. List of significant proteins from Differentially Expressed Proteins in sputum of 3-component remission in U-BIOPRED**

After conducting our differential protein expression on 3-component remission, we extracted significant proteins on two main thresholds: log_2_(FC) > 0.5 or < -0.5; unadjusted p-value <0.05. We present the proteins with log_2_(FC), unadjusted p-values and adjusted p-values with Benjamini-Hochberg correction. Analysis was only conducted on 3-component remission as no genetic data was available on the 3 patients who satisfied criteria for 4-component remission.

*ROC: Reciever Operating Characteristics; AUROC: Area Under Receiver Operating Characteristics curve; PPV: Positive Predictive Value; NPV: Negative Predictive Value; F1: F1 score*

**Supplementary Tables S5. List of significantly upregulated pathways** (based on DEGs at a *‘p < 0.05’*)

After conducting our differential gene expression on ATLANTIS’s 3-component remission, and 4-component remission, and differential protein expression in U-BIOPRED’s 3-component remission, we extracted upregulated genes and proteins on two main thresholds: log_2_(FC) > 0.5; unadjusted p-value < 0.05. Significantly upregulated genes and proteins were inserted in Reactome to undergo pathway enrichment analysis. We present the most significant pathways with p-value and False Discovery Rate. Although we consider a p < 0.05 to be significant for a pathway, we report and discuss all pathways extracted.

(a) represents ATLANTIS pathways in nasal brushings; (b) represents U-BIOPRED pathways in sputum

*FCGR3A: Fc gamma receptor IIIa; IL10: Interleukin 10; MITF-M: Microphthalmia-associated transcription factor-melanocyte; ESR: Endoplasmic Smooth Reticulum; NFEFL2: Nuclear factor erythroid 2-related factor 2; TGFBR2: Transforming Growth Factor Beta Receptor 2; TGFBR1: Transforming Growth Factor Beta Receptor 1; BMP: Bone Morphogenic Protein; Ca2+: Calcium 2+ ion; TGF-beta: Transforming Growth Factor-beta*

**Supplementary Tables S6. List of significantly downregulated pathways** (based on DEGs at a *‘p < 0.05’*)

After conducting our differential gene expression on ATLANTIS’s 3-component remission, and 4-component remission, and differential proteomic analysis in U-BIOPRED’s 3-component remission we extracted downregulated genes and proteins on two main thresholds: log_2_(FC) < -0.5; unadjusted p-value < 0.05. Significantly downregulated genes and proteins were inserted in Reactome to undergo pathway enrichment analysis. We present the most significant pathways with p-value and False Discovery Rate. Although we consider a p < 0.05 to be significant for a pathway, we report and discuss all pathways extracted.

(a) represents ATLANTIS pathways in nasal brushings; (b) represents U-BIOPRED pathways in sputum

*RAB: Rab proteins; GEF: Guanine nucleotide exchange factor; GTP: Guanosine Triphosphate; GPD: Guanosine diphosphate; WDR5: WD repeat-containing protein 5; PKMT: Protein Lysine Methyltransferases; RNX1: Runt related Transcription Factor 1; Ca2+: Calcium 2+; VEGF: Vascular Endothelial Growth Factor; TFAP2: Transcription Factor AP-2 alpha; VEGFR: Vascular Endothelial Growth Factor Receptor; IGFBPs: Insulin Growth Factor Binding Proteins; NGF: Nerve Growth Factor*

**Supplementary Tables S7. List of significantly upregulated and downregulated pathways** (based on DEGs at a *‘p < 0.01’*)

After conducting our differential gene expression on ATLANTIS’s 3-component remission, and 4-component remission we extracted upregulated and downregulated genes on a second more stringent p-value threshold to account for the lack of significant genes after FDR correction: unadjusted p-value < 0.01. DEGs were inserted in Reactome to undergo pathway enrichment analysis. We present the most significant pathways with p-value and False Discovery Rate. Although we consider a p < 0.05 to be significant for a pathway, we report and discuss all pathways extracted. In ATLANTIS, as only one DEG was significant at a p < 0.01, 3 pathways were produced.

(a) represents ATLANTIS upregulated pathways; (b) represents ATLANTIS downregulated pathways

*mRNA: messenger RNA; RNA: Ribonucleic acid; RAC3: Rac Family Small GTPase 3; GTPase: Guanosine Triphosphatase; SLIT: Slit protein; ROBO: Roundabout receptor GTP: Guanosine Triphosphatase*

**Supplementary Figure 1. U-BIOPRED Consort diagram**

We used a replication cohort in adults (U-BIOPRED).

*LDA: Low Disease Activity; DGE: Differential Gene Expression*

**Supplementary Figure 2** Correlation between PRCP gene expression and Low Disease Activity groups in the U-BIOPRED cohort. 0 = non-remission; 1 = remission

| **Variable** | **GVIF** | **Df** | **GVIF^(1/(2*Df))** |
| --- | --- | --- | --- |
| Age at diagnosis | 2781.678 | 1 | 52.742 |
| Age | 1543.32 | 1 | 39.285 |
| BMI | 1.146 | 1 | 1.07 |
| Sex | 1.06 | 1 | 1.03 |
| Number of exacerbations | 1.065 | 1 | 1.032 |
| Disease duration | 1877.097 | 1 | 43.325 |
| SADT Score | 1.018 | 1 | 1.009 |
| SAD Physiological Score | 1.571 | 1 | 1.254 |
| Post bronchodilator FEV_1_% predicted | 1.41 | 1 | 1.187 |
| GINA severity | 1.198 | 2 | 1.046 |
| FeNO (ppb) | 1.171 | 2 | 1.04 |
| Blood eosinophil count (×10^9^ L^−1^) | 1.199 | 2 | 1.046 |

**Supplementary Table 8**. Assessment of multicollinearity among model covariates using generalised variance inflation factors (GVIF). Values are presented as GVIF and GVIF^(1/(2×Df)). Higher values indicate greater collinearity. Age, age at diagnosis, and disease duration show substantial collinearity, reflecting their inherent interdependence, whereas all other variables demonstrate low collinearity.

**References**

1.	McNulty W, Usmani OS. Techniques of assessing small airways dysfunction. *Eur Clin Respir J* 2014; **1**.

2.	Kocks J, van der Molen T, Voorham J, et al. Development of a tool to detect small airways dysfunction in asthma clinical practice. *Eur Respir J* 2023; **61**(3).

3.	Postma DS, Brightling C, Baldi S, et al. Exploring the relevance and extent of small airways dysfunction in asthma (ATLANTIS): baseline data from a prospective cohort study. *Lancet Respir Med* 2019; **7**(5): 402-16.

4.	Szalma S, Koka V, Khasanova T, Perakslis ED. Effective knowledge management in translational medicine. *J Transl Med* 2010; **8**: 68.

5.	RStudio Team. RStudio: Integrated Development for R. RStudio, PBC, Boston, MA. http://www.rstudio.com/ [Accessed May 2, 2024].

6.	Love MI, Huber W, Anders S. Moderated estimation of fold change and dispersion for RNA-seq data with DESeq2. *Genome Biol* 2014; **15**(12): 550.

7.	Revelle W, Condon DM. Reliability from α to ω: A tutorial. *Psychol Assess* 2019; **31**(12): 1395-411.

8.	Fabregat A, Jupe S, Matthews L, et al. The Reactome Pathway Knowledgebase. *Nucleic Acids Res* 2018; **46**(D1): D649-d55.

9.	Dobin A, Davis CA, Schlesinger F, et al. STAR: ultrafast universal RNA-seq aligner. *Bioinformatics* 2013; **29**(1): 15-21.

10.	Reactome. Signaling by Interleukins . https://reactome.org/content/detail/R-HSA-449147.

11.	Reactome. Interleukin-4 and Interleukin-13 signaling. https://reactome.org/content/detail/R-HSA-6785807.

12.	Reactome. Intrinsic Pathway of Fibrin Clot Formation. https://reactome.org/content/detail/R-HSA-140837.

13.	Reactome. Formation of Fibrin Clot (Clotting Cascade) . https://reactome.org/content/detail/R-HSA-140877.
